# Carotenoid biosynthesis is associated with low-temperature adaptation in *Rhodosporidium kratochvilovae*

**DOI:** 10.1186/s12866-022-02728-2

**Published:** 2022-12-24

**Authors:** Rui Guo, Tao Liu, Caina Guo, Gongshui Chen, Jingdie Fan, Qi Zhang

**Affiliations:** Faculty of Life Science and Technology, Kunming University of Science and Technology, Kunming, 650500 China

**Keywords:** *Rhodosporidium kratochvilovae*, Low-temperature adaptation, Carotenoids, CRISPR/Cas9 gene-editing system, Lycopene cyclase/phytoene synthase gene

## Abstract

**Background:**

Low temperatures greatly limit the growth of microorganisms. Low-temperature adaptation in microorganisms involves multiple mechanisms. Carotenoids are naturally occurring lipid-soluble pigments that act as antioxidants and protect cells and tissues from the harmful effects of free radicals and singlet oxygen. However, studies on the regulation of carotenoid biosynthesis at low temperatures in microorganisms are limited. In this study, we investigated the correlation between carotenoids and low-temperature adaptation in the cold-adapted strain of *Rhodosporidium kratochvilovae* YM25235.

**Results:**

Carotenoid biosynthesis in YM25235 was inhibited by knocking out the bifunctional lycopene cyclase/phytoene synthase gene (*RKCrtYB*) using the established CRISPR/Cas9 gene-editing system based on endogenous U6 promoters. The carotenoids were extracted with acetone, and the content and composition of the carotenoids were analyzed by spectrophotometry and HPLC. Then, the levels of reactive oxygen species (ROS) and the growth rate in YM25235 were determined at a low temperature. The results indicated that the carotenoid biosynthesis and ROS levels were increased in the YM25235 strain at a low temperature and inhibition of carotenoid biosynthesis was associated with higher ROS levels and a significant decrease in the growth rate of YM25235 at a low temperature.

**Conclusions:**

The regulation of carotenoid biosynthesis was associated with low-temperature adaptation in YM25235. Our findings provided a strong foundation for conducting further studies on the mechanism by which YM25235 can adapt to low-temperature stress.

**Supplementary Information:**

The online version contains supplementary material available at 10.1186/s12866-022-02728-2.

## Background

Unicellular organisms need to maintain internal stability during fluctuations in the external environment. These environmental factors include temperature stress, osmolarity fluctuations, pH changes, UV irradiation, nutrient deprivation, drought, salt stress, etc. [1]. Organisms often acclimate to such stress conditions by producing appropriate levels of reactive oxygen species (ROS), which might, in turn, alter metabolism and induce programmed cell death [2]. Although ROS production is critical for growth, signaling, and development, the reactivity of ROS acts as a double-edged sword as a high level of ROS can damage cells. Antioxidant defense systems in cells and organelles, such as mitochondria and peroxisomes, protect against these toxic oxygen intermediates [3]. Enzymatic antioxidant systems counteract stress-induced ROS accumulation and include various scavengers, such as superoxide dismutase (SOD), ascorbate peroxidase (APX), glutathione peroxidase (GPX), glutathione S-transferase (GST), and catalase (CAT). Another type of antioxidant system consists of non-enzymatic low molecular metabolites, which include ascorbate (ASH), reduced glutathione (GSH), α-tocopherol, flavonoids, and carotenoids [4, 5].

Low temperatures cause abiotic stress; nearly 80% of the biosphere is under a permanent cold spell [6]. Low temperatures might disrupt microbial homeostasis and alter the functioning of cells [7]. Low temperatures can severely inhibit fungal growth and might even cause death under certain circumstances [8]. Under low-temperature stress, microorganisms might increase the levels of unsaturated fatty acids and modulate membrane flexibility [6, 9, 10]. Moreover, the biosynthesis of compatible solutes, membrane transporters, and antifreeze proteins is altered at low temperatures to enhance cryoprotection [11]. The content and composition of carotenoids are closely associated with ambient temperature. For example, the carotenoid content and composition of several species of red yeast (*Rhodotorula glutinis*, *R. mucilaginosa*, and *R. gracilis*) changed after they were cultured at a low temperature [12]. However, whether these changes are associated with alterations in the ROS levels in the cells needs to be determined.

Carotenoids are colored fat-soluble pigments present in plants, microorganisms, and animals, and their pigment colors range from yellow to red [13–15]. Carotenoid biosynthesis in yeast cells can be enhanced by changing the conditions of the culture environment [12], which include stressful conditions associated with changes in the temperature, visible or ultraviolet light irradiation, increase in the osmotic pressure (e.g., high concentrations of sodium chloride, sugars, and glycerol), presence of toxic substances (e.g., heavy metal salts, phenol, and methylene blue), and oxidative stress [16–21].

In the cytoplasm, carotenoids act as antioxidants and protect cells from free radicals and singlet oxygen [22]. They also exist in microbial cell membranes and affect cell membrane fluidity, which, in turn, is associated with adaptation to environmental stress [23]. Jagannadham et al. [24] found that the relative levels of carotenoids in *Sphingobacterium multivorum* were higher in the cultures at 5 °C compared to that in the cultures at 25 °C. Kot et al. [12] also found that carotenoid biosynthesis was higher at a low temperature (20 °C). However, the relationship between an increase in the biosynthesis of carotenoids and low temperatures needs further investigation.

*Rhodosporidium kratochvilovae* strain YM25235 (isolated from Chenghai Lake, Yunnan, China) is a cold-adapted strain of oleaginous yeast, which can grow in temperatures as low as 15 °C. In another study on the YM25235 strain, we found that low-temperature adaptation in the YM25235 strain was associated with polyunsaturated fatty acids (PUFAs), glycerol, and a two-component system in this strain [10, 25, 26]. In another study on the genome-wide transcriptional changes in the YM25235 strain at a low temperature, we found an association between carotenoids and low-temperature adaptation in the YM25235 strain [27]. We also found that carotenoid biosynthesis increases in the YM25235 strain at a low temperature (unpublished). However, the relationship between carotenoids and low-temperature adaptation in YM25235 needs further investigation.

## Results

### Prediction of the U6 promoters for sgRNA transcription

**Fig. 1:**
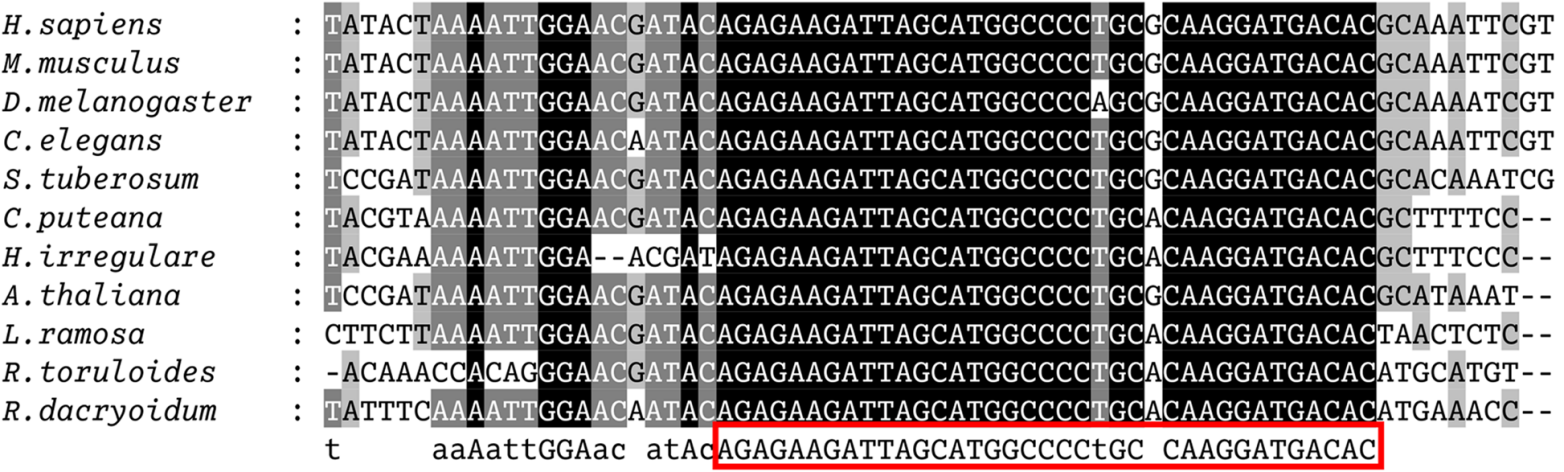
Alignment of the U6 genes from 11 organisms. The highly conserved region is indicated in red box

**Fig. 2:**
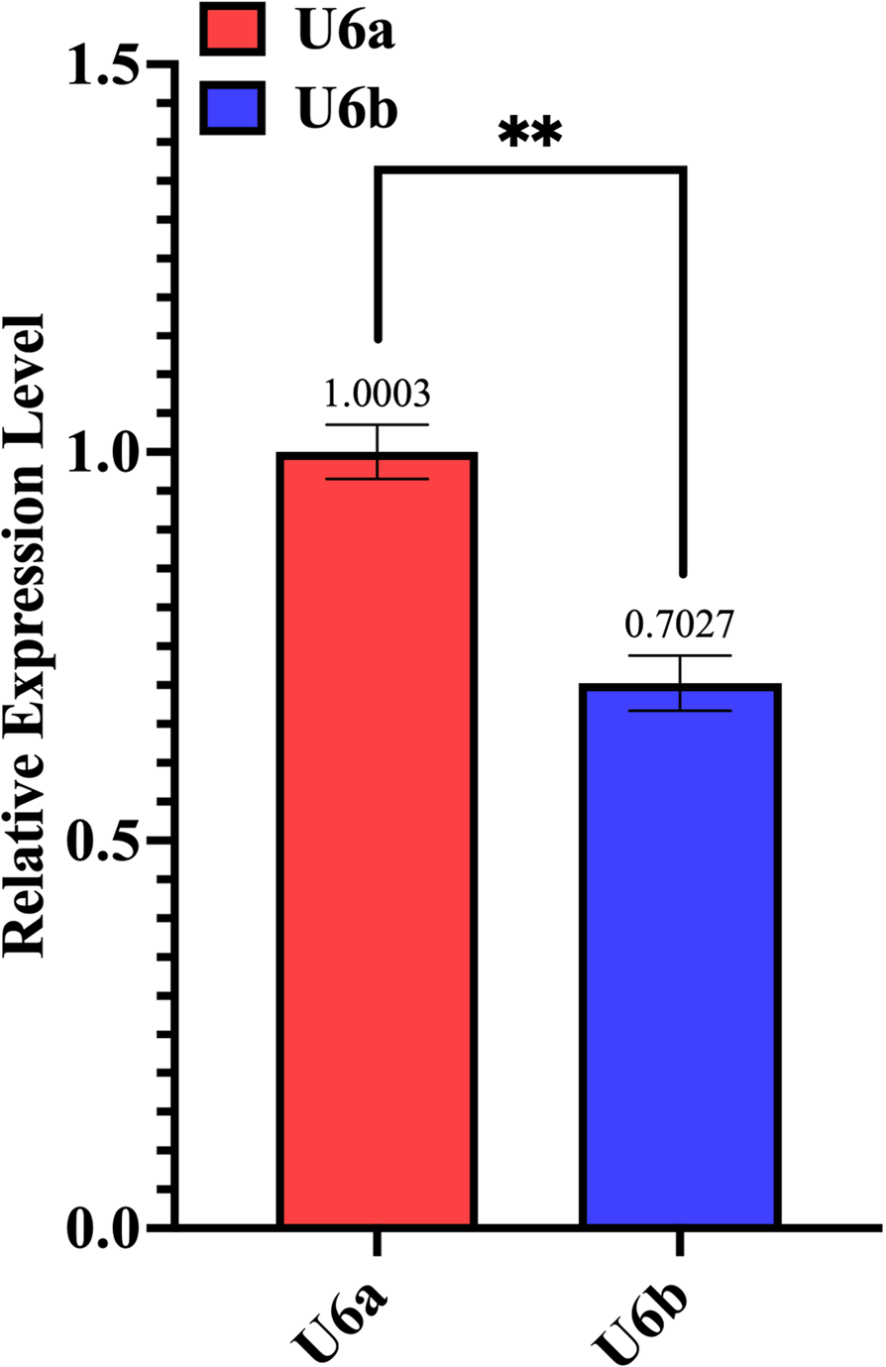
The sgRNA levels produced by transcription of the two promoters, as determined by the qPCR analysis. qPCR performed in triplicate; **p < 0.01

To identify the U6 promoters in the genome of YM25235, we compared the U6 gene sequences from 11 organisms and found a highly conserved region of approximately 50 bp (Fig. 1). The subsequent blastn analysis of this conserved region revealed two endogenous U6 genes distributed at different positions on contig 13 (U6a: 607,226–607,194; U6b: 614,275–614,307). Several studies have shown that the mature U6 RNA of *Rhodosporidium* sp. is approximately 110 bp long, and its transcription is initiated by the RNA polymerase III promoter at a purine base [28]. Based on the method described by Jiao et al. [29], the promoter fragments approximately 250 bp upstream of the labeled guanine were selected as the promoters U6a and U6b (Supplementary Table S1). The transformants corresponding to these promoters (U6a and U6b) were selected randomly and analyzed via qPCR. The results indicated that mature sgRNA was produced from both promoters. The U6a promoter produced higher levels of sgRNA (29.75%) than the U6b promoter (Fig. 2). Therefore, U6a was selected as the promoter for expressing sgRNA in subsequent experiments.

### Cas9 expression in the *Rhodosporidium kratochvilovae* strain YM25235

**Fig. 3:**
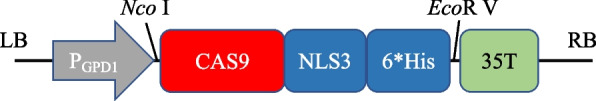
The schematic diagram of the Cas9-expression cassette. The synthesized codon-optimized 6*His-NLS-fused Cas9 was inserted the *Nco* I and *Eco*R V sites of the vector pRH034 under the control of the GPD promoter (pGPD1)

**Fig. 4:**
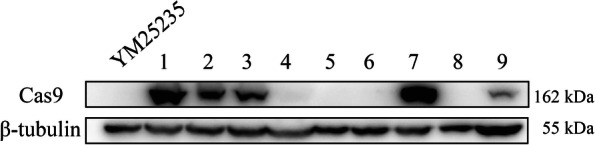
The expression of Cas9 (via the 6*His tag) was detected by the Western blotting analysis. Nine transformants of YM25235-Cas9 were selected for analysis. Among them, five transformants (lanes 1, 2, 3, 7, and 9) showed the expression of Cas9 protein, but the other four transformants (lanes 4, 5, 6, and 8) did not. The YM25235 strain was used as the negative control of Cas9 expression. The protein expression was normalized to β-tubulin expression

To implement the CRISPR/Cas9 system in YM25235, Cas9-expressed YM25235 hosts were first developed. The pRH2034 plasmid was used as the backbone for Cas9 expression (Fig. 3), and this plasmid was used for transforming the YM25235 strain. Next, the Western blotting analysis of the cell lysates of nine transformants was performed. The results showed that 6*His-NLS-fused Cas9 (~ 162 kDa) was expressed (Supplementary Table S2) in five transformants: YM25235-Cas9–1, YM25235-Cas9–2, YM25235-Cas9–3, YM25235-Cas9–7, and YM25235-Cas9–9 (Fig. 4 and Supplementary Fig. S1). The transformant YM25235-Cas9–7 exhibited the highest relative expression levels of Cas9 and was used in subsequent experiments, designated as YM25235-Cas9.

### Target mutagenesis of *RKCrtYB*

**Fig. 5:**
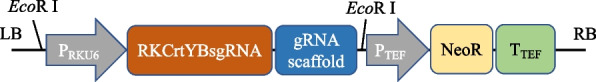
The schematic diagram of the U6 promoters for the expression of sgRNA. The U6 promoter, sgRNA and gRNA scaffold were inserted the *Eco*R I site of the vector pRG2304

**Fig. 6:**
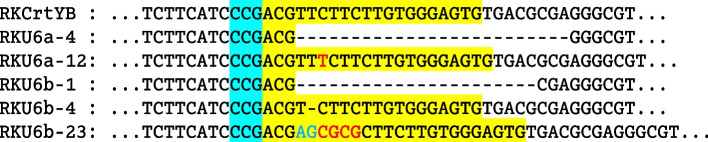
Sequence alignment of the DNA fragments obtained from the *RKCrtYB* gene of YM25235 with the DNA fragments obtained from the five albino transformants. The gRNA guiding sequence (yellow background), PAM sequence (light blue background), insertions (red), deletions (black dashes), and substitutions (blue) are indicated

To confirm the transcription of sgRNA, the PAM sequence and an NGG nucleotide sequence were identified at the 3′-end of the 20-bp genome target (Supplementary Table S3) in the target gene *RKCrtYB* (GenBank Access ID: ON768808). The plasmid for the transcription of sgRNA (Fig. 5) was constructed using the plasmid pRG2034 and integrated into the Cas9-expressing YM25235 strain YM25235-Cas9. When cultured on plates containing YPD supplemented with hygromycin and G418 sulfate, albino colonies were produced. To confirm that the mutation had occurred at the target locus, genomic DNA samples were prepared from the transformants and amplified by PCR using the primer pair YB1 and YB2 (Supplementary Table S4). The PCR products were sequenced, and the results showed that various deletion mutations occurred in the target locus of *RKCrtYB* (Fig. 6). A transformant was selected for the subsequent analyses and designated as YM25235/*RKCrtYB*Δ.

### Content and composition of carotenoids at a low temperature

To determine the carotenoid biosynthesis levels in the YM25235 strain at moderate (30 °C) and low (15 °C) temperatures and understand the effect of knocking out the *RKCrtYB* gene on carotenoid biosynthesis in the YM25235 strain, the carotenoid content in the YM25235 strain and the YM25235/*RKCrtYB*Δ strain were evaluated when cultured at 30 °C and 15 °C for 24 h. The results indicated that low-temperature treatment significantly increased carotenoid biosynthesis in the YM25235 strain. The most prominent increase occurred in torularhodin and β-carotene. The carotenoid content in the YM25235/*RKCrtYB*Δ strain obtained by knocking out the *RKCrtYB* gene using the CRISPR/Cas9 system was significantly lower than that in the YM25235 strain, and the contents of the four main carotenoid compositions in the YM25235/*RKCrtYB*Δ strain were undetected in the HPLC analysis. Moreover, the differences in the carotenoid content between the culture temperatures of 30 °C and 15 °C were not significantly different in the YM25235/*RKCrtYB*Δ strain (Table 1).

**Table 1:** The effect of low-temperature treatment on the content and composition of carotenoids in the two YM25235 strains

Strain	Tm (°C)	Carotenoid content (mg/g DCW)	Carotenoid composition (mg/g DCW)			
			Torularhodin	Torulene	γ-carotene	β-carotene
YM25235	30	2.48 ± 0.06	0.35 ± 0.01	0.20 ± 0.00	0.37 ± 0.01	1.41 ± 0.03
	15	3.17 ± 0.12**	0.54 ± 0.02**	0.22 ± 0.01	0.29 ± 0.01	2.03 ± 0.08**
YM25235/ *RKCrtYB*Δ	30	0.14 ± 0.01***	–	–	–	–
	15	0.13 ± 0.01	–	–	–	–

**p < 0.01, ***p < 0.001, compared to YM25235 at 30 °C

### ROS levels at a low temperature

**Fig. 7:**
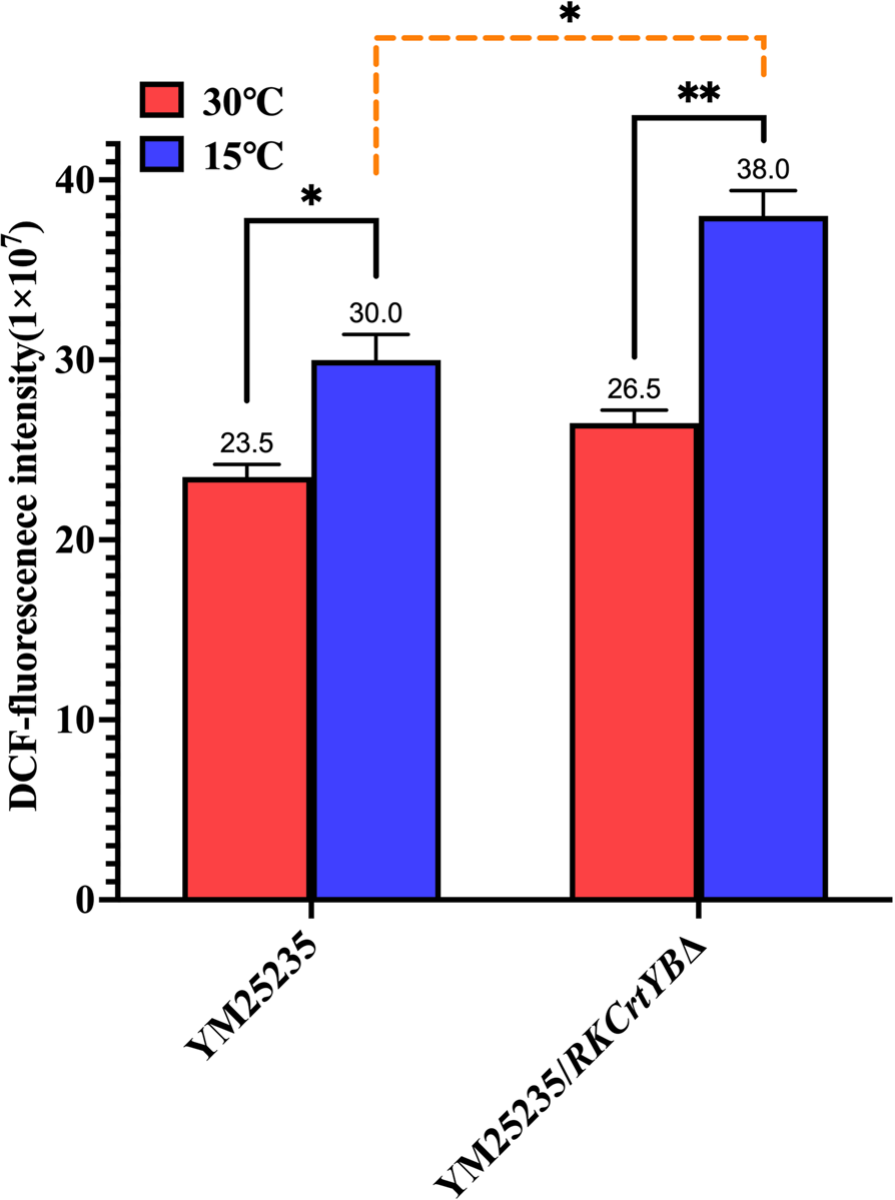
The effect of the regulation of carotenoid biosynthesis on the ROS levels in the two YM25235 strains at a low temperature. Red and blue bars indicate strains cultured at 30 °C and 15 °C, respectively; *p < 0.05 and **p < 0.01

The ROS levels were evaluated in the YM25235 strain and the YM25235/*RKCrtYB*Δ strain, which were cultured at moderate (30 °C) and low (15 °C) temperatures for 24 h. The results indicated that the ROS levels increased with a decrease in the culture temperature. For the YM25235 strain, the ROS level at 15 °C was 27.7% higher than that at 30 °C, while for the YM25235/*RKCrtYB*Δ strain, the ROS level at 15 °C was 43.4% higher than that at 30 °C. Additionally, at 15 °C, the ROS level in the YM25235/*RKCrtYB*Δ strain was significantly higher than that in the YM25235 strain (Fig. 7). To summarize, the ROS level increased in the YM25235 strain at a low temperature, but higher ROS level were observed in the YM25235/*RKCrtYB*Δ strain at the same low temperature. These results indicated that carotenoid biosynthesis could be helpful to inhibit the increase of ROS in the YM25235 strain at a low temperature.

### Growth of YM25235 at a low temperature

**Fig. 8.**
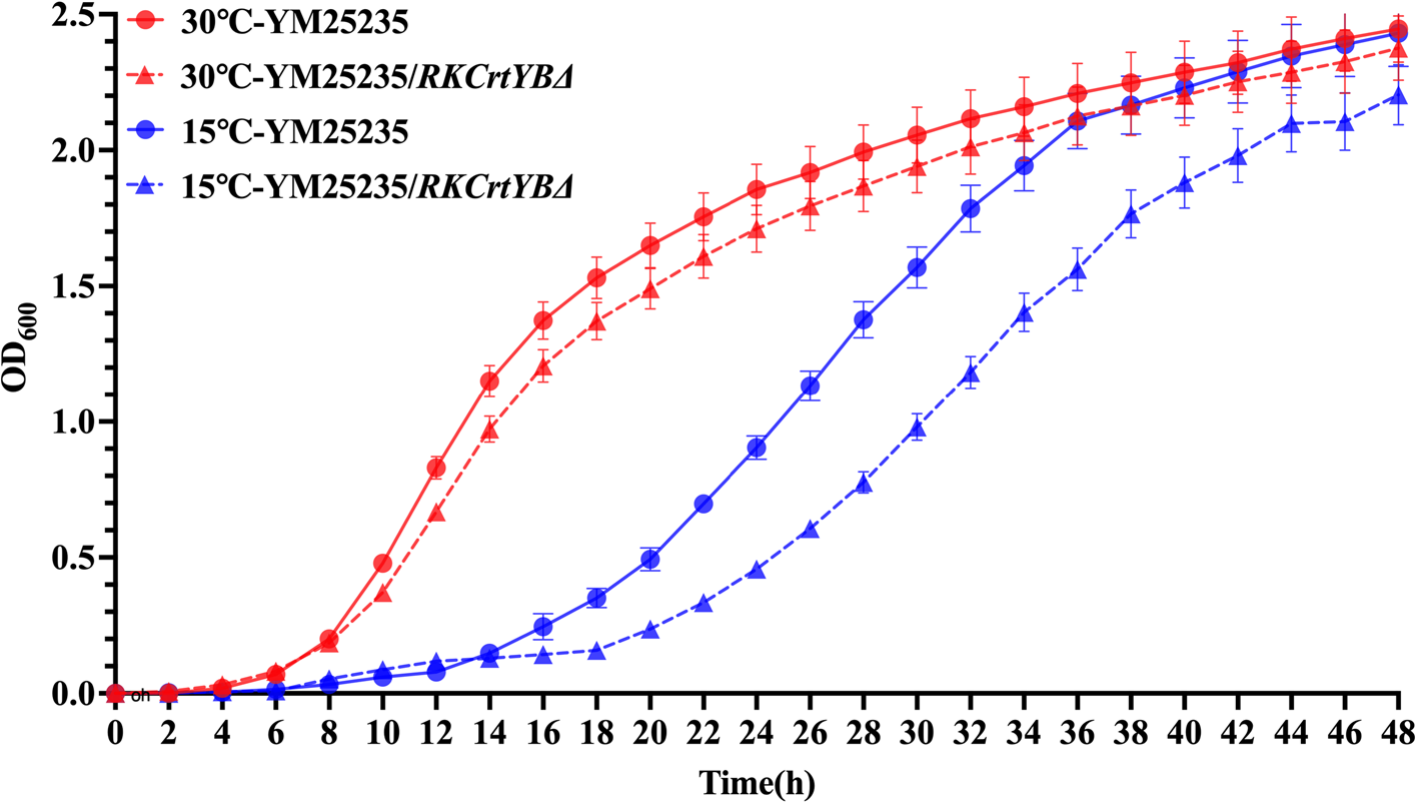
The effect of the regulation of carotenoid biosynthesis on the growth of the two YM25235 strains at a low temperature. The YM25235 strain (red line) and the YM25235/*RKCrtYB*Δ strain (blue line) were cultured at 30 °C (circles) and 15 °C (triangles), respectively

The growth of the YM25235 and YM25235/*RKCrtYB*Δ strains, cultured at moderate (30 °C) and low (15 °C) temperatures, was analyzed. The growth of both strains was lower at 15 °C than at 30 °C. Additionally, at 15 °C, the YM25235/*RKCrtYB*Δ strain did not grow as well as the YM25235 strain. However, at 30 °C, the two strains showed similar growth (Fig. 8). These results indicated that the YM25235 strain could adapt and grow well at a low temperature through the regulation of carotenoid biosynthesis.

## Discussion

Organisms commonly encounter low-temperature stress. Low temperatures affect cell growth, metabolic activity, energy production, and growth rate [30, 31]. The *R. kratochvilovae* strain YM25235 is an oil-producing red yeast adapted to low temperatures. We had previously shown that the partial regulatory mechanisms of the biosynthesis of PUFAs and glycerol in YM25235 at low temperatures were associated with the low-temperature adaptation of this strain. We also found that the level of carotenoid and trehalose biosynthesis increased significantly at a low temperature. Therefore, low-temperature adaptation in YM25235 might involve multiple mechanisms [25–27]. However, whether an increase in the level of carotenoid biosynthesis at low temperatures is associated with low-temperature adaptation in YM25235 remains to be investigated.

Carotenoids are naturally occurring lipid-soluble pigments. The C40 terpenoids are the most prevalent carotenoids present in nature. Carotenoid pigments occur universally in the photosynthetic systems of higher plants, algae, and phototrophic bacteria [32]. In non-photosynthetic organisms, carotenoids protect against photo-oxidative damage. Therefore, carotenoids are used by several non-phototrophic bacteria and fungi for protection against abundant light and air [33]. Studies on the biosynthesis of carotenoids at low temperatures in microorganisms, including the red yeast *Rhodosporidium*, are limited. Hayman et al. [34] showed that temperature affects the enzyme activity involved in carotenoid production, thus controlling the carotenoid levels in microorganisms. In this study, the *RKCrtYB* gene, which plays a key role in carotenoid biosynthesis in YM25235, was knocked down. The results indicated that knocking down this gene significantly inhibited carotenoid biosynthesis, which led to a decrease in low-temperature adaptation in YM25235. This finding indicated that carotenoid biosynthesis is associated with low-temperature adaptation in YM25235.

Reactive oxygen species play a dual role in organisms. High concentrations of ROS cause severe oxidative damage to DNA, RNA, proteins, and cellular membranes. They also act as important second messengers that induce several signaling cascades at low concentrations [35]. The relationship between ROS levels and low-temperature stress is well-understood. Low temperatures induce the accumulation of large quantities of intracellular ROS, which increases oxidative stress [36]. El-Agamey et al. [37] showed that carotenoids scavenge free radicals in three steps, which include electron transfer, hydrogen abstraction, and addition. Rutz et al. [38] showed that the presence of conjugated double bonds enables compounds to accept electrons from reactive species, which causes the neutralization of free radicals. Kot et al. [12] found that oxidative stress can increase the carotenoid content in several red yeasts. We found similar results in this study, i.e., the ROS levels and the total carotenoid content in the YM25235 strain increased at a low temperature. Therefore, we speculated that the ROS levels were responsible for the changes in the carotenoid content in YM25235, which then reduced the cellular damage caused due to the generation of ROS.

Generally, carotenoids are hydrophobic molecules with low solubility in water. Therefore, they function in the hydrophobic regions of the cell. The polarity of carotenoids is altered when polar functional groups are attached to the polyene chain, which might influence the localization of carotenoids within biological membranes and their interactions with other molecules [39]. Vershinin [40] reported that in primitive organisms, carotenoids reinforced lipids in the cell membrane. The study also inferred that the levels of carotenoids controlled the stability of the membrane (which is liable to change with temperature) and, thus, affected the functions of membranes. Carotenoids are also associated with photosynthetic membranes in certain fungi for protecting the cells against the light. They also protect organisms, including fungi, against oxidative stress [41]. However, the findings of this study did not confirm the relationship between carotenoid biosynthesis at low temperatures and the membranes in YM25235; thus, further studies are required to elucidate the relationship.

Besides affecting the total carotenoid content, growth temperature also affects the relative percentages of β-carotene, torulene, and torularhodin in carotenoid-producing yeasts. A study showed that the production of β-carotene and torulene increased while that of torularhodin decreased in *Rhodosporidiobolus colostri* under low-temperature conditions [42]. An increase in the production of β-carotene in *Rhodosporidium babjevae* (Golubev) was also found to occur under low-temperature treatment [43]. These findings indicated that the effect of temperature on the carotenoid composition is different in different strains, which could be because non-polar carotenoids, such as β-carotene, do not bind to the lipid head group [44]. This might increase the effectiveness of non-polar β-carotene in improving cell membrane fluidity compared to the effectiveness of polar components [45]. In this study, the production of β-carotene in YM25235 increased significantly at a low temperature, which was similar to the findings of other studies. However, the production of torularhodin also increased in YM25235 at a low temperature. This inconsistency might be due to the differences between the strains or the different culture condition requirements of the strains. The composition of carotenoids might change with an increase in the duration of exposure to low temperatures.

Technological advancements have considerably increased the scope of manipulating and studying microorganisms [46]. We used the homologous recombination (HR) technology to knock out genes, although the extremely low HR frequency of YM25235 and the complex experimental manipulations made it challenging to knock out genes in this organism. The CRISPR/Cas9 system has revolutionized the genetic engineering process for several organisms [47] and might also solve the above-mentioned challenges. Using this technology, only a 20 nucleotide guide RNA (gRNA) sequence needs to be changed to introduce precise cuts into most loci in the genome using the Cas9 enzyme. Thus, this technology can be used for targeted genetic manipulations without the requirement of markers [48]. Furthermore, unlike previous technologies and methods, the CRISPR-Cas9 technology can be used to obtain gene edits in a few days [49]. Moreover, this technology uses NHEJ to create site-specific gene deletions [50], which provides greater advantages. However, although the CRISPR/Cas9 technology is extensively used for engineering microbial genomes and editing targeted nucleotides without DSBs, its application in non-model microbes, including red yeasts, is limited. Also, the strong species specificity of the U6 promoter, an important component of the CRISPR/Cas9 system, limits the application of this system to only a few species [51]. In a study, the sgRNA levels produced using the U6 promoter in *R. toruloides* were significantly higher (> 200-fold) than those produced using the U6 promoter from *Saccharomyces cerevisiae* in the CRISPR/Cas9 system of *R. toruloides* [29]. Therefore, the CRISPR/Cas9 systems constructed for a particular species are generally not optimized for other species. Jiao et al. [29] and Schultz et al. [52] have reported CRISPR/Cas9 systems for the *R. toruloides* strain NP11; however, a CRISPR/Cas9 system for *R. kratochvilovae* is not known*.* In this study, a CRISPR/Cas9 system was constructed and used as a novel genome-editing tool for the *R. kratochvilovae* strain YM25235 for the first time. The CRISPR/Cas9 system might be used for accelerating metabolic engineering efforts in *R. kratochvilovae* in future studies. In this study, we used the CRISPR/Cas9 system to determine the relationship between carotenoids and low-temperature adaptation in the *R. kratochvilovae* strain YM25235 by knocking out the *RKCrtYB* gene. The results showed that carotenoid biosynthesis was associated with low-temperature adaptation in the *R. kratochvilovae* strain YM25235.

## Conclusion

In this study, we found that carotenoid biosynthesis was associated with low-temperature adaptation in the YM25235 strain by knocking out *RKCrtYB* (a key gene for carotenoid biosynthesis) using a CRISPR/Cas9 gene editing system. The findings of this study provided vital information that might be used in future studies to determine the mechanisms by which YM25235 can adapt to low temperatures. Understanding these mechanisms can contribute to the production of bioactive molecules from YM25235.

## Materials and methods

### Strains and media

The samples of *R. kratochvilovae* strain YM25235 were collected from Chenghai Lake, Yunnan, China, and stored. They were revived at 30 °C in the yeast extract peptone dextrose [YPD: 1% yeast extract, 2% peptone, and 2% glucose] culture medium. The culture medium (YPD) was supplemented with 30 mg/L of G418 sulfate or 150 mg/L of hygromycin B if required. The *Escherichia coli* strain DH5α was cultured in the LB medium [1% NaCl, 1% peptone, and 0.5% yeast extract] at 37 °C.

### Designing the sgRNA

To design the sgRNA for genome editing, the sgRNAs targeting nine exon-exon junctions in the *RKCrtYB* gene were selected using CRISPOR (http://crispor.tefor.net/). The sequence motif 5′-N(20)-NGG-3′ was identified. Since the sgRNA scaffold needs to be complementary to prevent potential off-target sites from being targeted [53], the selected protospacer was searched using the blastn program in the genome of the *R. kratochvilovae* strain YM25235 [27]. Finally, one sequence motif with high CRISPOR scores and no off-target sites was selected for constructing the sgRNA transcription vectors.

### Plasmid construction

To construct the recombinant Cas9 protein-expressing plasmid pRHCas9, the codon-optimized Cas9 gene from *Streptococcus pyogenes* was synthesized by Tsingke Biotechnology Co. Ltd. (Beijing, China). The synthesized codon-optimized Cas9 was then digested with *Nco* I and *Eco*R V and subcloned into the vector pRH2304 [54] to replace the *GFP* gene. To construct the sgRNA transcription plasmids, the gRNA scaffold and the designed sgRNA were synthesized by Tsingke Biotechnology Co. Ltd. (Beijing, China), and the promotors P_RKU6a_ and P_RKU6b_ from the genomic DNA of the YM25235 strain were amplified using the primer pairs U6a-F/U6a-R and U6b-F/U6b-R (Supplementary Table S4). The sequences of the U6a-sgRNA-gRNA scaffold and the U6b-sgRNA-gRNA scaffold were subcloned using the ClonExpress MultiS One-Step Cloning Kit (Vazyme, Nanjing, China) into the vector pRG2304, which was previously digested with *Eco*R I. All constructs were verified through digestion with the respective restriction enzymes and DNA sequencing.

### Yeast transformation and screening

The YM25235 strain was transformed using the constructed plasmid pRHCas9, as described in another study [26]. The positive transformants of the yeast were selected on a YPD agar plate containing 150 μg/mL of hygromycin B. The transformants were then identified by performing PCR and Western blotting analyses to obtain the recombinant Cas9 protein-expressing strain YM25235-Cas9. The YM25235-Cas9 strain was further transformed with the sgRNA transcription plasmids, as described in another study [26]. The positive transformants were then selected on a YPD agar plate containing 30 mg/L of G418 sulfate and 150 μg/mL of hygromycin B.

### Western blotting

Western blotting was performed to detect protein expression. The samples were prepared and subjected to sodium dodecyl sulfate-polyacrylamide gel electrophoresis (SDS-PAGE), as described in another study [26].

### Real-time quantitative polymerase chain reaction

The sgRNA transcription analysis was performed using 5 mL of the sample cultured for 24 h and harvested at 4 °C. Total RNA extraction, and quantitative PCR (qPCR) analyses were conducted as described in another study [26].

### Carotenoid content and composition analysis

The weight of the dry cells was quantified in triplicate for each sample. Briefly, the culture samples were centrifuged at 5000 rpm for 5 min, followed by washing the cell pellets twice with distilled water. The washed cell pellets were dried in an oven at 50 °C until a constant weight was achieved (after approximately 18 h). To extract carotenoids from the cell pellets, the dried cell pellet samples were ground and extracted with acetone at a solid-liquid ratio of 1:5. The mixture was shaken vigorously and centrifuged at 13,000 rpm for 5 min. The extraction process was repeated thrice, followed by filtration and sample preparation for the spectrophotometry analysis of the carotenoid content and HPLC analysis of the carotenoid composition.

### Determining ROS levels

The level of ROS in the cells was measured following the instructions provided in the Reactive Oxygen Species Assay Kit (Beyotime Institute of Biotechnology; Haimen, China). The non-fluorescent probe 2′,7′-dichlorofluorescein diacetate (H_2_DCF-DA) was allowed to diffuse into the cells, where it was deacetylated to form non-fluorescent 2′,7′-dichlorofluorescein (DCFH). DCFH then reacted with the ROS in the cells to generate the fluorescent product DCF, which remained trapped inside the cells. Fluorescence in the cells was detected at 485 nm excitation and 530 nm emission using a fluorescence microplate reader. An increase in the fluorescence intensity relative to the fluorescence intensity in control indicated an increase in the intracellular levels of ROS.

### Yeast growth analysis

Each activated strain was cultured in the YPD medium at 30 °C and 15 °C in triplicate. The samples were retrieved every 2 h for 48 h. From the retrieved samples, the OD_600_ values of the yeast fluid were measured using a UV–VIS spectrophotometer.

### Statistical analysis

All results were analyzed statistically using the GraphPad Prism 9 software (GraphPad Software, CA). The data were expressed as the mean ± standard deviation (SD). The parameters between groups were compared by performing the unpaired Student’s t-test. All differences between groups were considered to be statistically significant at *p* < 0.05.

## Supplementary Information

**Additional file 1:** **Supplementary Table S1.** The sequences of promoter PRKU6a and promoter PRKU6b. **Supplementary Table S2.** The sequence of codon-optimized Cas9-NLS-6*His. **Table S3.** The sequence of sgRNA of RKCrtYB used in the present study. **Supplementary Table S4.** Primers used in the present study.

**Additional file 2:** **Figure S1.** The original image of the expression of Cas9 detected in the western blotting analysis (Figure 4).

## Data Availability

The datasets generated and/or analyzed during the current study are available in the GenBank repository (https://www.ncbi.nlm.nih.gov/genbank/, accession ON768808).
