# Supplementary material for: Carotenoid biosynthesis is associated with low-temperature adaptation in Rhodosporidium kratochvilovae
Source: BMC Microbiol. 2022 Dec 24;22:319. doi: 10.1186/s12866-022-02728-2 (PMC9789556; doi:10.1186/s12866-022-02728-2)
Supplement: Supplementary file 1 — Additional file 1: Supplementary Table S1. The sequences of promoter PRKU6a and promoter PRKU6b. Supplementary Table S2. The sequence of codon-optimized Cas9-NLS-6*His. Table S3. The sequence of sgRNA of RKCrtYB used in the present study. Supplementary Table S4. Primers used in the present study. [file 12866_2022_2728_MOESM1_ESM.docx]

**Carotenoid biosynthesis is associated with low-temperature adaptation in *Rhodosporidium kratochvilovae***

Rui Guo^1,2^, Tao Liu^1^, Caina Guo^1^, Jingdie Fan^1^ and Qi Zhang^1*^

1 Faculty of Life Science and Technology, Kunming University of Science and Technology, Kunming 650500, China.

2 School of Life Sciences and Technology, Tongji University, Shanghai 200120, China.

*Correspondence: Qi Zhang, qzhang37@kust.edu.cn

Supplementary Table S1. The sequences of promoter P_RKU6a_ and promoter P_RKU6b_

| **Name** | **Sequence** |
| --- | --- |
| promoter P_RKU6a_ | TCTGTTCAGTCCATGTAGCTCGTCAGTAAGCGCCAAGCCAGCTCTTCGAGTCGGTCGAACTGGGACGGACACCTCGCTGAGCCGGGACGAGGGAGGAGACGGGTTACAAGGTGCCACAGCCCACCTCGCCATCCACGCTCAAATGCTACCGTTTCTTGCTCGCGCTGCTTCGTACTTTCTGCGATCGTCAAAACCATCCACCCTACGGGGTGTGATATACATAAAATTGGAACGATACAGGCGAGCTCCT |
| promoter P_RKU6b_ | CTCCGTTCAGTCTGTGCAGCTAGCCATTCAGCGCCAACCCAGTTCTTCGAGTTGGTCGAGCTGGAACGGACACCTCGCTGAGCCGGGACGAGGTAGCAGCTGGGCTACAAGCCGCGACAACCTGCCTCGCCGTCCGCGCTCAAATGCTACCGTTTCTTGTTTGCACTGGTTTGTACTTTCTGCGATCGTCAAAACCATCCACCCTACGGGGTGTGATATACATAAAATTGGAACGATACAGGCGAGCTCC |

Supplementary Table S2. The sequence of codon-optimized Cas9-NLS-6*His.

| **Name** | **Sequence** |
| --- | --- |
| 6*His-NLS-Cas9 | ATGGACAAGAAGTACTCGATCGGCCTCGACATCGGCACCAACTCGGTCGGCTGGGCCGTCATCACCGACGAGTACAAGGTCCCGTCGAAGAAGTTCAAGGTCCTCGGCAACACCGACCGCCACTCGATCAAGAAGAACCTCATCGGCGCCCTCCTCTTCGACTCGGGCGAGACCGCCGAGGCCACCCGCCTCAAGCGCACCGCCCGCCGCCGCTACACCCGCCGCAAGAACCGCATCTGCTACCTCCAGGAGATCTTCTCGAACGAGATGGCCAAGGTCGACGACTCGTTCTTCCACCGCCTCGAGGAGTCGTTCCTCGTCGAGGAGGACAAGAAGCACGAGCGCCACCCGATCTTCGGCAACATCGTCGACGAGGTCGCCTACCACGAGAAGTACCCGACCATCTACCACCTCCGCAAGAAGCTCGTCGACTCGACCGACAAGGCCGACCTCCGCCTCATCTACCTCGCCCTCGCGCACATGATCAAGTTCCGCGGCCACTTCCTCATCGAGGGCGACCTCAACCCCGACAACTCGGACGTCGACAAGCTCTTCATCCAGCTCGTCCAGACCTACAACCAGCTCTTCGAGGAGAACCCGATCAACGCCTCGGGCGTCGACGCCAAGGCCATCCTCTCGGCCCGCCTCTCGAAGTCGCGCCGCCTCGAGAACCTCATCGCCCAGCTCCCCGGCGAGAAGAAGAACGGCCTCTTCGGCAACCTCATCGCGCTCTCGCTCGGCCTCACCCCCAACTTCAAGTCGAACTTCGACCTCGCCGAGGACGCCAAGCTCCAGCTCTCGAAGGACACCTACGACGACGACCTCGACAACCTCCTCGCCCAGATCGGCGACCAGTACGCCGACCTCTTCCTCGCCGCCAAGAACCTCTCGGACGCCATCCTCCTCTCGGACATCCTCCGCGTCAACACCGAGATCACCAAGGCCCCCCTCTCGGCCTCGATGATCAAGCGCTACGACGAGCACCACCAGGACCTCACCCTCCTCAAGGCCCTCGTCCGCCAGCAGCTCCCCGAGAAGTACAAGGAGATCTTCTTCGACCAGTCGAAGAACGGCTACGCCGGCTACATCGACGGCGGCGCCTCGCAGGAGGAGTTCTACAAGTTCATCAAGCCGATCCTCGAGAAGATGGACGGCACCGAGGAGCTCCTCGTCAAGCTCAACCGCGAGGACCTCCTCCGCAAGCAGCGCACCTTCGACAACGGCTCGATCCCGCACCAGATCCACCTCGGCGAGCTCCACGCCATCCTCCGCCGCCAGGAGGACTTCTACCCGTTCCTCAAGGACAACCGCGAGAAGATCGAGAAGATCCTCACCTTCCGCATCCCCTACTACGTCGGCCCGCTCGCGCGCGGCAACTCGCGCTTCGCCTGGATGACCCGCAAGTCGGAGGAGACCATCACCCCCTGGAACTTCGAGGAGGTCGTCGACAAGGGCGCCTCGGCCCAGTCGTTCATCGAGCGCATGACCAACTTCGACAAGAACCTCCCGAACGAGAAGGTCCTCCCGAAGCACTCGCTCCTCTACGAGTACTTCACCGTCTACAACGAGCTCACCAAGGTCAAGTACGTCACCGAGGGCATGCGCAAGCCGGCCTTCCTCTCGGGCGAGCAGAAGAAGGCCATCGTCGACCTCCTCTTCAAGACCAACCGCAAGGTCACCGTCAAGCAGCTCAAGGAGGACTACTTCAAGAAGATCGAGTGCTTCGACTCGGTCGAGATCTCGGGCGTCGAGGACCGCTTCAACGCCTCGCTCGGCACCTACCACGACCTCCTCAAGATCATCAAGGACAAGGACTTCCTCGACAACGAGGAGAACGAGGACATCCTCGAGGACATCGTCCTCACCCTCACCCTCTTCGAGGACCGCGAGATGATCGAGGAGCGCCTCAAGACGTACGCCCACCTCTTCGACGACAAGGTCATGAAGCAGCTCAAGCGCCGCCGCTACACGGGCTGGGGCCGCCTCTCGCGCAAGCTCATCAACGGCATCCGCGACAAGCAGTCGGGCAAGACCATCCTCGACTTCCTCAAGTCGGACGGCTTCGCCAACCGCAACTTCATGCAGCTCATCCACGACGACTCGCTCACCTTCAAGGAGGACATCCAGAAGGCCCAGGTCTCGGGCCAGGGCGACTCGCTCCACGAGCACATCGCCAACCTCGCCGGCTCGCCGGCCATCAAGAAGGGCATCCTCCAGACCGTCAAGGTCGTCGACGAGCTCGTCAAGGTCATGGGCCGCCACAAGCCGGAGAACATCGTCATCGAGATGGCCCGCGAGAACCAGACCACCCAGAAGGGCCAGAAGAACTCGCGCGAGCGCATGAAGCGCATCGAGGAGGGCATCAAGGAGCTCGGCTCGCAGATCCTCAAGGAGCACCCCGTCGAGAACACCCAGCTCCAGAACGAGAAGCTCTACCTCTACTACCTCCAGAACGGCCGCGACATGTACGTCGACCAGGAGCTCGACATCAACCGCCTCTCGGACTACGACGTCGACCACATCGTCCCCCAGTCGTTCCTCAAGGACGACTCGATCGACAACAAGGTCCTCACCCGCTCGGACAAGAACCGCGGCAAGTCGGACAACGTCCCCTCGGAGGAGGTCGTCAAGAAGATGAAGAACTACTGGCGCCAGCTCCTCAACGCCAAGCTCATCACCCAGCGCAAGTTCGACAACCTCACCAAGGCCGAGCGCGGCGGCCTCTCGGAGCTCGACAAGGCCGGCTTCATCAAGCGCCAGCTCGTCGAGACCCGCCAGATCACCAAGCACGTCGCCCAGATCCTCGACTCGCGCATGAACACCAAGTACGACGAGAACGACAAGCTCATCCGCGAGGTCAAGGTCATCACCCTCAAGTCGAAGCTCGTCTCGGACTTCCGCAAGGACTTCCAGTTCTACAAGGTCCGCGAGATCAACAACTACCACCACGCCCACGACGCCTACCTCAACGCGGTCGTCGGCACCGCCCTCATCAAGAAGTACCCGAAGCTCGAGTCGGAGTTCGTCTACGGCGACTACAAGGTCTACGACGTCCGCAAGATGATCGCCAAGTCGGAGCAGGAGATCGGCAAGGCCACCGCCAAGTACTTCTTCTACTCGAACATCATGAACTTCTTCAAGACCGAGATCACGCTCGCCAACGGCGAGATCCGCAAGCGCCCCCTCATCGAGACCAACGGCGAGACCGGCGAGATCGTCTGGGACAAGGGCCGCGACTTCGCCACCGTCCGCAAGGTCCTCTCGATGCCCCAGGTCAACATCGTCAAGAAGACCGAGGTCCAGACCGGCGGCTTCTCGAAGGAGTCGATCCTCCCGAAGCGCAACTCGGACAAGCTCATCGCCCGCAAGAAGGACTGGGACCCGAAGAAGTACGGCGGCTTCGACTCGCCCACGGTCGCCTACTCGGTCCTCGTCGTCGCCAAGGTCGAGAAGGGCAAGTCGAAGAAGCTCAAGTCGGTCAAGGAGCTCCTCGGCATCACCATCATGGAGCGCTCGTCGTTCGAGAAGAACCCGATCGACTTCCTCGAGGCGAAGGGCTACAAGGAGGTCAAGAAGGACCTCATCATCAAGCTCCCGAAGTACTCGCTCTTCGAGCTCGAGAACGGCCGCAAGCGCATGCTCGCCTCGGCCGGCGAGCTCCAGAAGGGCAACGAGCTCGCCCTCCCGTCGAAGTACGTCAACTTCCTCTACCTCGCCTCGCACTACGAGAAGCTCAAGGGCTCGCCCGAGGACAACGAGCAGAAGCAGCTCTTCGTCGAGCAGCACAAGCACTACCTCGACGAGATCATCGAGCAGATCTCGGAGTTCTCGAAGCGCGTCATCCTCGCCGACGCGAACCTCGACAAGGTCCTCTCGGCCTACAACAAGCACCGCGACAAGCCCATCCGCGAGCAGGCCGAGAACATCATCCACCTCTTCACCCTCACCAACCTCGGCGCCCCGGCCGCCTTCAAGTACTTCGACACCACCATCGACCGCAAGCGCTACACCTCGACCAAGGAGGTCCTCGACGCCACCCTCATCCACCAGTCGATCACCGGCCTCTACGAGACCCGCATCGACCTCTCGCAGCTCGGCGGCGACGAGATCCGCGAGCTCAAGCGCCGCCTCGGCGAGTGCGAGGCCGGCGACGCCGGCACCCGCAAGCGCGTCAAGTACGAGCACCACCACCACCACCACTAG |

Table S3. The sequence of sgRNA of *RKCrtYB* used in the present study.

| **Name** | **Sequence** |
| --- | --- |
| RKCrtYBsgRNA | ACGTTCTTCTTGTGGGAGTG |

Supplementary Table S4. Primers used in the present study.

| **Name** | **Sequence** |
| --- | --- |
| **Promoters P_RKU6a_ and P_RKU6b_ Amplification** | |
| U6a-F | TCTGTTCAGTCCATGTAGCT |
| U6a-R | AGGAGCTCGCCTGTATC |
| U6b-F | CTCCGTTCAGTCTGTGCAGC |
| U6b-R | GGAGCTCGCCTGTATCGTTC |
| **Sequencing of gRNA Target Sites** | |
| YB1 | GACCTACCTCGCCCTTATCG |
| YB2 | GGCCGTTGAGGTATATCGCG |
