# Supplementary material for: Carotenoid biosynthesis is associated with low-temperature adaptation in Rhodosporidium kratochvilovae
Source: BMC Microbiol. 2022 Dec 24;22:319. doi: 10.1186/s12866-022-02728-2 (PMC9789556; doi:10.1186/s12866-022-02728-2)
Supplement: Supplementary file 2 — Additional file 2: Figure S1. The original image of the expression of Cas9 detected in the western blotting analysis (Figure 4). [file 12866_2022_2728_MOESM2_ESM.docx]

**Carotenoid biosynthesis is associated with low-temperature adaptation in *Rhodosporidium kratochvilovae***

Rui Guo, Tao Liu, Caina Guo, Gongshui Chen, Jingdie Fan and Qi Zhang^*^

1 Faculty of Life Science and Technology, Kunming University of Science and Technology, Kunming 650500, China.

2 School of Life Sciences and Technology, Tongji University, Shanghai 200120, China.

*Correspondence: Qi Zhang, qzhang37@kust.edu.cn

**
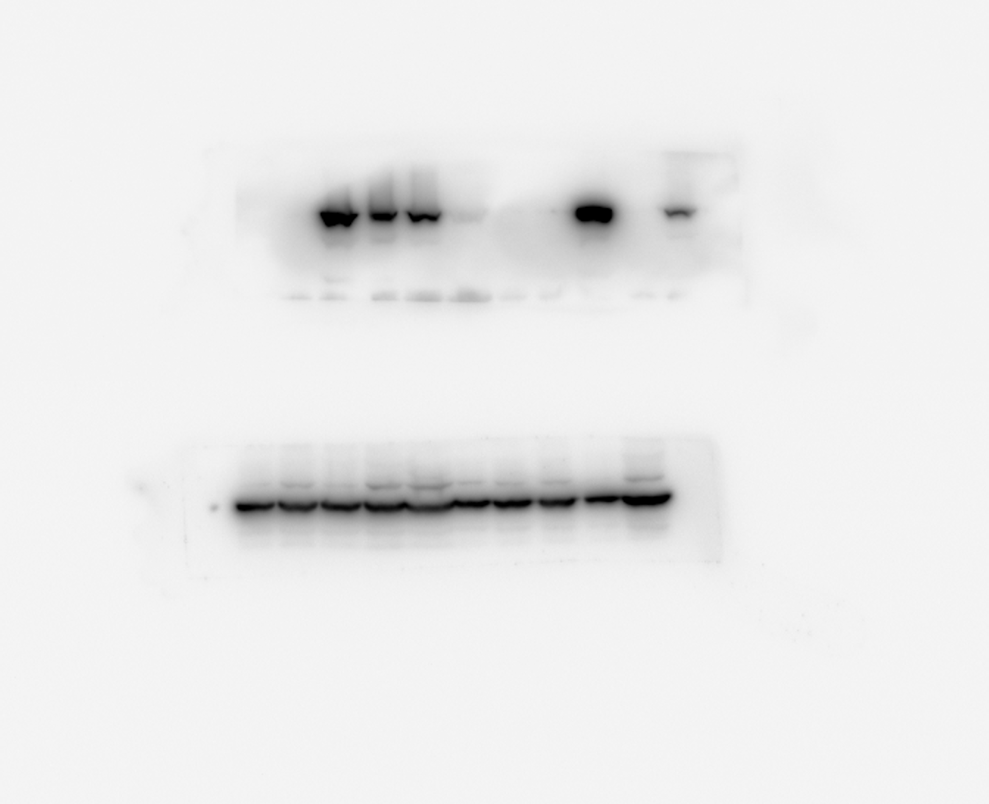
**

**Figure S1**. The original image of the expression of Cas9 detected in the western blotting analysis (Figure 4).
